# The effect of mindfulness-based stress reduction therapy on maternal anxiety, depression, and sleep quality: A protocol for systematic review and meta-analysis

**DOI:** 10.1097/MD.0000000000028849

**Published:** 2022-02-25

**Authors:** Zhongrong Chen, Jianmei Jiang, Tingting Hu, Lan Luo, Cheng Chen, Wei Xiang

**Affiliations:** The Central Hospital of Enshi Tujia and Miao Autonomous Prefecture, Enshi, Hubei Province, China.

**Keywords:** anxiety, depression, maternal, meta-analysis, mindfulness-based stress reduction, protocol, sleep

## Abstract

**Background:**

Depression and anxiety are common in the prenatal and postnatal periods, which significantly influence pregnant women and their unborn babies. Pharmacological interventions can negatively affect maternal and infant health outcomes, while psychotherapy can avoid adverse events of medication and improve maternal depression and anxiety. Whether mindfulness-based stress reduction (MBSR) can alleviate maternal anxiety and depression and improve sleep quality is still controversial. Therefore, we aim to conduct a meta-analysis by collecting randomized controlled trials (RCTs) reporting the effects of MBSR on maternal anxiety, depression, and sleep quality, thus providing evidence-based medical evidence for non-pharmacological interventions.

**Methods:**

RCTs reporting the effect of MBSR on maternal anxiety, depression, and sleep quality versus conventional obstetric care will be searched in online databases, including the Cochrane Central Register of Controlled Trials Repositories, PubMed, Embase, Web of Science, Chinese Science Citation Database, China National Knowledge Infrastructure, Chinese Biomedical Literature Database, Chinese Scientific Journal Database, and Wan Fang Database. Literature selection, data extraction, risk of bias assessment, and meta-analyses will be independently completed by 2 researchers. Meta-analysis will be performed by using RevMan5.4.

**Results:**

The results of this meta-analysis will be submitted to a peer-reviewed journal for publication.

**Conclusion:**

This study will provide reliable evidence-based evidences for the effects of MBSR on improving maternal anxiety, depression, and sleep quality.

## 1 Introduction

In recent years, maternal mental health has been well concerned due to the increased awareness of pregnancy care. Pregnancy and childbirth are special periods in a woman's life. The high psychological stress and negative emotions such as anxiety and depression due to concerns about the health of the fetus, fear of labor pain, and lack of proper understanding of pregnancy and childbirth may increase the risk of postpartum depression.^[1]^ It is shown that about 10% to 15% of women suffer from anxiety or depression during the 12 months after delivery.^[2]^ Up to 10% of women have a severe traumatic stress to childbirth, and 3% develop post-traumatic stress disorder after delivery.^[3]^ Nearly 64% of new mothers feel fatigued and stressed after childbirth.^[4]^ Lack of a timely intervention would harm women's health newborn development. Therefore, effective interventions to alleviate maternal depression and anxiety have attracted a widespread attention.

Mindfulness therapy has been introduced and gradually applied to alleviate various psychological disorders such as anxiety and depression, showing a remarkable efficacy. It has also been applied in interpersonal communication, personality disorders, and impulse control.^[5–7]^ Mindfulness-based stress reduction (MBSR) is effective in relieving psychological stress by awakening the inner concentration and improving self-regulation through mindfulness meditation, body awareness, yoga, etc.^[8,9]^ It presents outstanding advantages than pharmacological interventions in relieving stress and enhancing beliefs.

Whether MBSR can relieve maternal anxiety, depression, and improve sleep quality remains unclear.^[10–17]^ This study aims to systematically analyze the effects of MBSR on maternal anxiety, depression, and sleep quality by investigating existing randomized controlled trials (RCTs), thus providing evidence-based medical evidence for non-drug interventions.

## 2 Methods

### 2.1 Protocol

This protocol of systematic review and meta-analysis was carried in adherence to preferred reporting items for systematic reviews and meta-analysis protocols (PRISMA-P) guidelines.^[18]^ The research framework has been registered on the open science framework (Registration Number: DOI 10.17605/OSF.IO/TK74H).

### 2.2 Ethics

As all data utilized in this systematic review and meta-analysis are published, ethical approval was not needed.

### 2.3 Eligibility criteria

#### 2.3.1 Types of studies

RCTs report the effect of MBSR on maternal anxiety, depression, and sleep quality without limitations in the published language.

#### 2.3.2 Types of participants

Pregnant women older than 18 years without limitations in the race, nationality, or duration of illness, who are volunteered to participate in will be analyzed. Those with psychiatric illness, bipolar disorder, substance abuse or dependence, psychotherapy, and severe depression or suicidal ideation will be excluded.

#### 2.3.3 Types of interventions

MBSR, including mindfulness-based cognitive therapy, meditation, body scan, and other routine care are given to pregnant women in experimental group.

#### 2.3.4 Types of outcome measurements

(1) Anxiety scores graded by the Self-Rating Anxiety Scale and Baker Anxiety Scale;
(2) Depression scores graded by the Edinburgh Postnatal Depression Scale, Beck Depression Inventory, and Self-Rating Depression Scale;
(3) Sleep quality assessed by the Pittsburgh sleep quality index.

### 2.4 Exclusion criteria

(1) Reviews, non-clinical studies, meta-analyses, etc;
(2) Full text is unavailable;
(3) Duplicate publications;
(4) Other psychological interventions are given to pregnant women in experimental group;
(5) Complete clinical data are unavailable.

### 2.5 Searching strategy

RCTs reporting the effect of MBSR on maternal anxiety, depression, and sleep quality versus conventional obstetric care published before January 2022 will be searched in online databases, including the Cochrane Central Register of Controlled Trials Repositories, PubMed, Embase, Web of Science, Chinese Science Citation Database, China National Knowledge Infrastructure, Chinese Biomedical Literature Database, Chinese Scientific Journal Database, and Wan Fang Database. In addition, references of eligible literatures will be manually reviewed. Searching strategy in the PubMed are summarized in Table 1.

**Table 1:** PubMed search strategy.

Number	Search terms
#1	Maternal[Title/Abstract]
#2	Pregnancy[Title/Abstract]
#3	Postpartum[Title/Abstract]
#4	Maternity[Title/Abstract]
#5	or/1–4
#6	Mindfulness-based stress reduction [Title/Abstract]
#7	MBSR[Title/Abstract]
#8	Mindfulness [Title/Abstract]
#9	Meditation[Title/Abstract]
#10	or/6–9
#11	Randomized Controlled Trials as Topic[MeSH]
#12	Clinical Trials, Randomized[Title/Abstract]
#13	Controlled Clinical Trials, Randomized[Title/Abstract]
#14	Trials, Randomized Clinical[Title/Abstract]
#15	Random^∗^[Title/Abstract]
#16	or/11-15
#17	#5 and #10 and #16

### 2.6 Data screening and extraction

**Figure 1:**
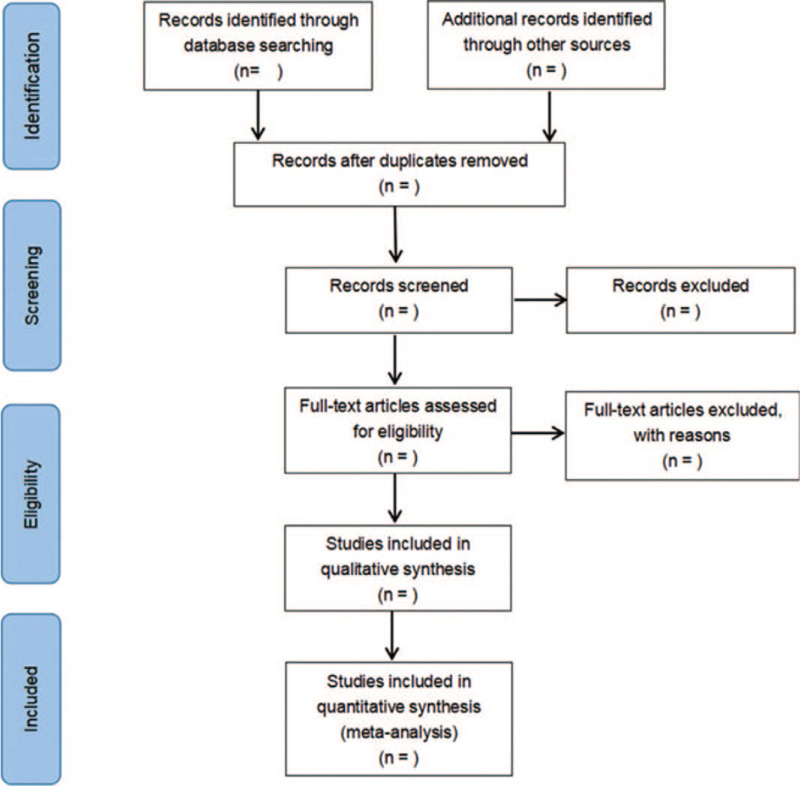
Flow diagram of literature retrieval.

Two investigators will independently screen the literature at each level according to the inclusion and exclusion criteria. Any disagreement will be solved by a mutual discussion. The following data will be extracted from eligible literatures: first author, year of publication, study area, health status of the study population, sample size, intervention, duration of intervention, and outcome indicators, etc. The literature selection process is listed in Figure 1.

### 2.7 Quality evaluation

The quality of the literature will be independently graded by 2 researchers according to the Cochrane Handbook recommended RCT risk of bias assessment tool on six items, including the method of random assignment, whether the assignment is concealed, whether blinding is used, whether the outcome data are complete, whether the findings are reported selectively, and whether there are other sources of bias.^[19]^ Each item will be graded into “low risk of bias,” “high risk of bias,” and “unclear.”

### 2.8 Statistical analysis

Statistical analysis will be performed using RevMan 5.3. The weighted mean difference (WMD) and corresponding 95% confidence interval (CI) of continuous data measured by the same method will be calculated, and the standardized mean difference (SMD) and corresponding 95%CI of those measured by different methods will be calculated. Heterogeneity between studies will be determined by the Chi-square test and *I*^2^ test will be used to determine the magnitude of heterogeneity. *P *≥* *.1 and *I*^2^* *≤* *50% are considered as a small heterogeneity and a fixed-effects model will be adopted; Otherwise, a random-effects model will be used to calculate the pooled data due to a large heterogeneity.

#### 2.8.1 Dealing with missing data

Insufficient or missing data in the literature will be obtained by e-mailing the authors. If data are still not available, only the current available data will be analyzed and the potential impacts will be discussed.

#### 2.8.2 Subgroup analysis

Subgroup analyses based on the age of all enrolled subjects, type of treatment, duration of treatment, and treatment in the control group will be performed.

#### 2.8.3 Sensitivity analysis

The robustness of the results will be tested by excluding studies with low quality and high risk of bias.

#### 2.8.4 Publication bias

If the number of included studies is no less than 10, a funnel plot will be drawn to assess publication bias.^[20,21]^

## 3 Discussion

A variety of factors contribute to maternal psychological problems.^[2–4,22]^ MBSR is a systematic meditation, in which mindfulness techniques are used to manage the emotions, cope with stress, and promote physical and mental health.^[23,24]^ Previous evidences have supported the effectiveness of MBSR on alleviating various chronic diseases.^[25–27]^ This study aims to comprehensively analyze the efficacy of MBSR on maternal anxiety, depression, and sleep quality interventions by a meta-analysis via collecting relevant RCTs.

## Author contributions

**Conceptualization:** Wei Xiang and Zhongrong Chen.

**Data collection:** Lan Luo and Cheng Chen.

**Data curation:** Wei Xiang, Jianmei Jiang.

**Formal analysis:** Jianmei Jiang.

**Funding acquisition:** Zhongrong Chen.

**Investigation:** Wei Xiang, Jianmei Jiang.

**Methodology:** Jianmei Jiang.

**Project administration:** Zhongrong Chen.

**Resources:** Wei Xiang, Jianmei Jiang, Lan Luo, Tingting Hu.

**Software:** Tingting Hu, Lan Luo, Jianmei Jiang.

**Supervision:** Lan Luo and Cheng Chen, Zhongrong Chen.

**Validation:** Tingting Hu, Cheng Chen.

**Visualization:** Tingting Hu, Cheng Chen.

**Writing – original draft:** Wei Xiang and Zhongrong Chen.

**Writing – review & editing:** Wei Xiang and Zhongrong Chen.
